# Occupational and Financial Setbacks in Caregivers of People with Colorectal Cancer: Considerations for Caregiver-Reported Outcomes

**DOI:** 10.3390/curroncol29110646

**Published:** 2022-10-29

**Authors:** A. Fuchsia Howard, Kelsey Lynch, Sally Thorne, Antony Porcino, Leah Lambert, Mary A. De Vera, Angela C. Wolff, Penelope Hedges, Scott M. Beck, María-José Torrejón, Mary T. Kelly, Michael McKenzie

**Affiliations:** 1 School of Nursing, The University of British Columbia, Vancouver, BC V6T 2B5, Canada; 2 BC Cancer, Vancouver, BC V5Z 4C2 Canada; 3 Faculty of Pharmaceutical Sciences, The University of British Columbia, Vancouver, BC V6T 1Z3, Canada; 4 School of Nursing, Trinity Western University, Langley, BC V2Y 1Y1, Canada; 5 Faculty of Medicine, The University of British Columbia, Vancouver, BC V6T 1Z3, Canada

**Keywords:** caregiver, family, supportive care needs, employment, occupation, financial costs, economic costs, qualitative research, caregiver reported outcomes, patient reported outcomes, colorectal cancer, oncology, psychosocial

## Abstract

Family caregivers of patients with cancer provide substantial physical, emotional, and functional care throughout the cancer trajectory. While caregiving can create employment and financial challenges, there is insufficient evidence to inform the development of caregiver-reported outcomes (CROs) that assess these experiences. The study purpose was to describe the occupational and financial consequences that were important to family caregivers of a patient with colorectal cancer (CRC) in the context of public health care, which represent potential considerations for CROs. In this qualitative Interpretive Description study, we analyzed interview data from 78 participants (25 caregivers, 37 patients, and 16 healthcare providers). Our findings point to temporary and long-term occupational and financial setbacks in the context of CRC. Caregiving for a person with CRC involved managing occupational implications, including (1) revamping employment arrangements, and (2) juggling work, family, and household demands. Caregiver financial struggles included (1) responding to financial demands at various stages of life, and (2) facing the spectre of lifelong expenses. Study findings offer novel insight into the cancer-related occupational and financial challenges facing caregivers, despite government-funded universal health care. Further research is warranted to develop CRO measures that assess the multifaceted nature of these challenges.

## 1. Introduction

There is growing acknowledgment that, in addition to physical, emotional, and practical consequences, cancer treatment and care impacts the finances of patients and their family caregivers [1,2,3]. Financial hardship can arise from direct out-of-pocket costs for treatment, which have escalated owing to high prices of newer classes of therapies and supportive medications, and indirect costs such as transportation and employment or occupation changes [4]. Financial consequences for patients can translate into emotional distress (anxiety and depression) [5], higher symptom burden [6], diminished quality of life [6,7,8], and reduced adherence to treatments and limited access to care [2]. For example, multiple American studies have reported that about half of cancer survivors experience some form of financial distress, with a significant proportion being in debt because of their treatment [2]. Some studies also found significant numbers did not adhere to prescribed medications because of costs [2]. There has been recognition that the financial burden is as important as physical toxicities, posing an often overlooked and serious consequence, with the term financial toxicity emerging to describe the financial burden and distress that can arise for patients and their family members [9,10].

Financial hardship is perhaps most acutely felt by patients and their families in jurisdictions with primarily privately funded health care, such as the United States, due to gaps in insurance coverage and higher costs of care. Yet, because financial hardship for patients in countries with government-funded universal health care has also been observed, it is recognized as a global issue affecting countries across health system structures and income levels [11,12,13,14,15]. While one might assume that publicly funded health care, such as in Canada, ensures equal access to cancer treatment for all in need with minimal costs incurred by patients, substantial cancer-related economic burdens can arise because not all aspects of direct care are fully funded, such as ambulatory medications, medical supplies, and limits on homecare or allied health care [11]. Moreover, there are often many indirect costs associated with such issues as occupational and employment disruptions for patients and their family caregivers. Policies and programs exist in most Canadian provinces to mitigate medical costs not covered by public funding, such as income replacement, tax breaks, and employer-provided health insurance. However, these policies and programs can be difficult to access because of complicated application processes and they are largely designed for patients and not caregivers. A 2021 Canadian study reported that 33% of 901 patients on active cancer treatment indicated a high burden of out-of-pockets-costs within the past 28 days and reported spending an average of 34% of their monthly income on cancer-related costs [11]. Further, 29% of patients took time off work, averaging 18 days off, while 26% indicated their family caregiver took time off work, averaging 11.5 days off over that same period. Qualitative analysis of patient comments on surveys describe financial burden stemming from costs incurred (associated with medications, food, transportation, and house-related costs particularly for those with functional decline) coupled with reduced income and financial reserves, contributing to financial difficulty and emotional distress [12].

Family caregivers of patients with cancer provide a substantial amount of physical, emotional, and functional care and support throughout the cancer trajectory. The economic costs of doing so are considerable because of the intensity of the care they provide and the cost and complexity of cancer treatment [16]. A 2016 American study reported that two-thirds of cancer caregivers averaged 33 h a week providing care, with half of those providing 41 or more hours of care per week [17]. In a review of 19 studies, care time, valued by measuring the time spent providing indirect care (i.e., time spent on activities of daily living, household activities or with the care recipient at the hospital or medical appointments), was the largest source of cancer caregiver costs [18]. Employed caregivers face the challenge of providing this care while continuing to meet work demands and pursue professional goals. Caregiver income and employment is often essential to cover family expenses, particularly in situations where the patient cannot work, or where the coverage of employment-based extended health insurance proves to be insufficient. Like patients, it is highly likely that caregivers experience emotional distress and reduced quality of life associated with financial burden, though studies have been rather limited to date [19,20].

Caregiver needs are often overlooked despite the considerable role that caregivers play as members of the cancer care team, the extent of caregiver burden, and the increased risk of caregiver morbidity and mortality [21,22]. It is also increasingly apparent that, to support cancer patients effectively, the needs of caregivers must also be understood and subsequently met [23]. Recognizing that the assessment of caregiver needs and outcomes is rarely a standard of care in oncology settings [12], our team sought to identify caregiver-reported outcomes (CROs) that reflect what is important to caregivers and to identify how the importance of these outcomes changes across the cancer trajectory. Like patient-reported outcomes, we conceptualized CROs as a caregiver’s assessment of their own health status and quality of life as a result of caring for a patient with cancer. We chose qualitative methodology to identify the outcomes that would be important from the perspective of caregivers and to ground the conceptualization and operationalization of CRO constructs and domains in caregivers’ experiences. 

We focused on caregivers of patients with colorectal cancer (CRC) because CRC is the third most commonly diagnosed cancer in Canada and the 5-year net survival for CRC is only 65% [24]. Patients often suffer a high physical symptom burden [25], multimodality therapy is required, prognosis is uncertain and poor particularly for metastatic disease [26], and family caregiver involvement is extensive. Further, the presence of a stoma for many patients has been found to significantly alter not only patient quality of life, sexuality and body image, and cause psychological morbidity but also to contribute dramatically to caregiver burden, including financial challenges [27,28]. While caregiver burden may dissipate over time and following completion of primary CRC treatment, considerable disruption has also been shown to persist for years owing to the ongoing nature of caregiving tasks and changes to a caregiver’s life [28]. 

In this large qualtiative research, conducted in Canada where there is government-funded universal health care, it became apparent that caregivers experienced occupational and financial consequences stemming from caring for a patient with CRC. Thus, the purpose of the analysis we describe here is to describe the occupational and financial consequences that stood out as important to caregivers throughout the cancer trajectory, and which represent potential considerations for the development and/or implementation of conceptually robust CRO measures.

## 2. Materials and Methods

This study was guided by Interpretive Description [29], an applied qualitative approach used to construct experiential evidence that is relevant to practice disciplines and clinical application. This was conducted as patient-oriented research [30] wherein patient- and caregiver-partners (individuals with lived experience), clinicians, and multidisciplinary stakeholders were equal research team members through all phases. That is, all research team members provided input into study protocol development, recruitment, the carrying out of data collection and analysis, and the dissemination of results. These patient- and caregiver-partners, clinicians, and multidisciplinary stakeholders were not study participants and were not interviewed during data collection. The protocol was approved by the harmonized BC Cancer and University of British Columbia Research Ethics Board. Further details of study methods have been published elsewhere [31,32].

### 2.1. Setting and Sample Recruitment

This research was conducted in British Columbia, Canada, where the government-funded health care system serves a population exceeding 5 million. Included caregiver participants were ≥19 years of age, spoke English and were involved in providing care to a relative (a spouse, unmarried partner, parent, sibling, adult child), a neighbour, or a friend with CRC. We did not stipulate a minimum level of caregiver involvement nor that the caregiver needed to be the primary family caregiver because we aimed to capture diversity of caregiver experiences and be inclusive of situations where caregiving was shared. Included patient participants were diagnosed with CRC, ≥19 years of age and spoke English. Participant recruitment involved distributing study information through the online newsletters and social media pages of caregiver and oncology organizations, and an email listserv of individuals who had consented to be contacted for research purposes. To secure maximal variation in the convenience sample and because of robust interest from self-selecting participants, we included more caregiver and patient participants than initially planned. 

We also recruited healthcare providers as study participants considering their insights as complementary to those of caregivers and patients and a source of triangulation. Included healthcare providers were those in acute or community settings who provided care to CRC patients. Healthcare providers were identified through the research team’s professional networks and emailed an invitation to participate. 

Though we used convenience sampling, we were guided by Interpretive Description [29] to continue to recruit participants until we had representation across caregiver and patient demographic (i.e., age, gender, relationship of caregiver to patient, living arrangements) and medical characteristics (i.e., patient cancer stage, patient with a colostomy or ileostomy). For healthcare providers we aimed for variation in type of provider. Furthermore, in line with Interpretive Description, we continued recruitment until we had robust variation in the information gleaned from participant interviews, a decision informed by the collective wisdom of the research team. Interpretive Description positions this approach to variation in sampling as an alternative to theoretical saturation sampling, which is continued sampling and data collection until no new insights emerge from the data.

### 2.2. Data Collection

In-depth, semi-structured participant interviews were conducted virtually via Zoom Video Communications, Inc. from April 2020 to November 2020 owing to restrictions on in-person research related to the COVID-19 pandemic. Caregiver and patient participants were given the option of doing interviews separately or together as a dyad (caregiver and patient interviewed together). Caregiver, patient, and healthcare provider interview guides included questions about priorities for CRO assessments, the communication of these assessments to healthcare providers and the patient, and recommendations for the implementation of CRO measures into routine practice. Examples of the questions posed to caregivers included: Tell me about the main challenges you struggled with as a caregiver when your loved one was first diagnosed. How were those challenges different from the challenges you faced during treatment? What about now (after treatment/near end-of-life)? How did the importance of those challenges change? What has helped you cope with the challenges you have faced? What factors (e.g., challenges and positive aspects of caregiving) would you like healthcare providers to ask you about? When would this be helpful/unhelpful to you? What would you like healthcare providers to do with this information? What type and when would support be helpful/unhelpful to you [31]? We asked patients similar questions in terms of what they perceived to be caregivers’ experiences and needs. Examples of questions posed to healthcare providers included: When and how do you assess caregiver challenges and needs in your practice? What factors do you think are most important to assess at the time of diagnosis, during treatment, and following treatment completion? How should these assessments be done? What would be helpful to support you in assessing caregivers’ challenges and needs? Interviews lasted 30–90 min, were audio recorded (not video-recorded), transcribed verbatim, de-identified, and checked for accuracy. Consistent with Interpretive Description, we focused on gathering interview data that was high in information power [33]. Information power indicates that the more information the sample holds relevant to the study, the fewer participants needed [33], and was enhanced in this study by the wide variation of in-depth accounts provided by study participants. That is, there was wide variation in the participants responses to questions posed in the interviews and in the nature of the experiences they described.

### 2.3. Data Analysis

A group of five team members involved in more in-depth analysis identified, discussed, and inductively developed an initial coding frame that included the very broad descriptive themes initially evident in the data, with occupational and financial challenges of caregiving being one. The application of the initial coding frame to the entire data set was completed by three team members using the data management software NVivo^TM^ Version 12 (QSR International, Burlington, MA, USA). We then harvested all data that we had broadly coded as occupational and financial challenges in the larger data set. Though the larger data set included information about the communication of CRO assessments and recommendations for CRO implementation in general, this was not the focus of the present analysis. Using an Interpretive Description approach [29], two team members then inductively identified patterns, diversities, and initial categories within the occupational and financial challenges data, which informed our development and application of a coding frame to the data. Two team members then compared and contrasted pieces of data within and across participants, a technique known as constant comparison [34]. The initial analysis was of the caregiver and patient interview data together and then the developing findings were compared to the healthcare provider interview data. The healthcare provider data aligned with and complemented the caregiver and patient data and, therefore, the occupational and financial challenges coding frame was also applied to these data. Two team members then grouped and regrouped the analytic categories, proceeding until we were confident that our conceptualization of the findings depicted the occupational and financial consequences that were experienced by, and important to, caregivers of a patient with CRC. During the process of writing the study findings and obtaining input from the larger research team, we were guided by Interpretive Description to intentionally aim for a higher level of conceptualization and to move from description to interpretation [29]. Notably, we centred the results on caregiver and patient accounts and included healthcare provider accounts when this extended or offered additional insight. 

## 3. Results

A total of 78 individuals (25 caregivers, 37 patients, and 16 healthcare providers) participated (see Table 1 for demographic details). We did not collect individual or household income data because this has been a sensitive question in our past qualitative research and we elected not to try to identify a proxy measure for comparative status. We did, however, seek a diversity in caregivers and patients reflecting a range of those who were employed versus not employed and of those employed, were working full-time versus part-time. Healthcare providers included 6 nurses, 1 nurse practitioner, 2 family physicians, 1 medical oncologist, 1 radiation oncologist, 2 social workers, 2 registered dietitians, and 1 genetic counsellor. 

Our findings point to temporary and long-term occupational and financial setbacks associated with CRC management. Caring for a person with CRC involved managing occupational implications, including (1) revamping employment arrangements, and (2) juggling work, family, and household demands. Caregiver financial struggles included (1) responding to financial demands at various stages of life, and (2) facing the spectre of lifelong expenses. 

### 3.1. Managing the Occupational Implications

#### 3.1.1. Revamping Employment Arrangements

The caregivers, patients, and healthcare providers described a plethora of adjustments caregivers made in their work and career lives to fulfill the responsibilities of caring for a partner or family member with CRC. Initially, caregivers negotiated vacation days, sick days, and then leaves of absence, but over time many caregivers realized that the strain of their responsibilities demanded they take extended time off work, forgo advancement, resign, or retire early. Many participants expressed the view it was impossible to work full-time and meet the expectations of being a caregiver. Caregiving required a workplace culture and direct manager that were flexible to deal with the many appointments and hospital trips. Many caregivers negotiated employment leaves, and some participants, such as this daughter, did so repeatedly:

*I struggled with being away from my family as well, and then also missing work and temporarily missing some income from work because I was taking leaves and things like that to help care for my mom…*

*(Participant 8)*

However, negotiating a leave was difficult for some. For example, after running out of sick days and vacation time, an anxious wife resorted to declaring mental health issues to procure more time off work saying, “there was no way I could be working and be worrying about him in the hospital” (Participant 57). 

Working remotely made caregiving easier for some. Like other study participants, one caregiver reflected that she would not have been able to continue working and act as primary caregiver without the flexibility arising from the option of working remotely (Participant 60). Similarly, a woman whose mother had advanced CRC described how her remote working situation allowed greater involvement in care with a move to her mother’s city of residence (Participant 64). For others, being self-employed made it possible to assume the caregiver role. However, this usually involved caregivers scaling back their paid work, as described by a self-employed daughter whose mother had CRC, but who also added that “if I were in a full-time job, it wouldn’t have been as easy to do that” (Participant 4). 

Even when caregivers successfully negotiated leaves or reduced their employment to part-time, the sole and prolonged focus on caregiving could be disruptive. For example, a woman who cared for her mother described that “a big part my identity is the work I do. I love the work I do… and so having to step away from that into the caregiving piece, to not have that aspect of my identity any longer was really challenging” (Participant 64). As evident in this quote, caregivers were faced with making choices between continuing their meaningful and successful careers or carrying out caregiver duties. When caregiving coincided with employment advancement, it was not always possible to request a leave, take vacation time or scale back their commitments. Thus, several participants described caregiver career advancement suffering and not recovering, as exemplified by a woman caring for a partner who finally gave up an opportunity after attempting to perform in a new position:

*I was writing for a [a publication] at the time and I was working from home. And I had taken on the role of editor-in-chief of [that publication]. So, I couldn’t take time off really. And I would work from the hospital and I would work at night… and that was tough… I quit my job in September of that year eventually, ‘cause I just couldn’t be there mentally anymore. So, I never got back to, to writing after that.*

*(Participant 50)*

The caregivers and patients commonly described caregivers’ attempts to reorganize and revamp their work lives, but in some instances that ultimately resulted in job loss. For some, this was framed as a reasonable choice and one that they felt privileged to be able to make within the circumstances of their lives. One man with CRC described his cousin this way:

*She attended every appointment, big or small, she was there and she just, she juggled, to make everything work… she just stopped [working in her profession]…*

*(Participant 21)*

However, other participants described the decision to quit their employment or occupation as extremely difficult, resulting in a sense of loss and grief. One wife shared her story:

*I had to give up my work life. So, that was a challenge, because, you know, I didn’t have any other outlet and I think that made it hard. I think that you just sort of get into routines, gotta go, okay, we’re going to the hospital, we’re going to the doctor, we gotta do this. You know, you get into that because I’m the driver… sometimes I go a little crazy because I think: where’s my life?*

*(Participant 58)*

In the case of several families, the loss of the caregiver’s employment was not a voluntary choice, but rather a consequence of performing caregiving roles and responsibilities. A wife who cared for her husband described how the initial decision to take a leave of absence ended up in job loss:

*I’m on caregiver leave. So, technically my company can’t get rid of my job, but I was back to work for about three months and they let me go because they knew that my husband wasn’t quite done… he would be having another surgery…. So, I would be pretty ridiculous if I didn’t make that connection.*

*(Participant 59)*

In the end, while some of the participants had the opportunity to completely revamp or give up their employment situation, the demands of caregiving were exceptionally difficult for employed family members and this constituted a significant concern to many. 

#### 3.1.2. Juggling Work, Family, and Household Demands 

The caregivers, patients and healthcare providers described caregivers juggling many responsibilities while caring for a loved one with CRC. A woman caring for her partner said: “I don’t know how everybody lives this. I wasn’t working anymore at the time and I was basically living at the hospital” (Participant 50). The day-to-day responsibilities were overwhelming for many caregivers, especially those who were employed, as exemplified by this woman who cared for her mother with CRC:

*I was swept into the day-to-day management and just getting through it and putting one foot in front of the other on what needs to be done next. I sort of went on to autopilot… I don’t feel that I was lacking in support, but it was just not something that crossed my mind because I was so absorbed in just the doing.*

*(Participant 4)*

Working caregivers with children were put in a tricky position, working, parenting, and maintaining their own households in addition to tending to the family member’s illness. A daughter caring for her mother with CRC described the endless juggling over time:

*[It] felt like a never-ending series of events. It seemed like, okay, we’re out of the woods and she was better. And then she got worse again. And, she needed another operation… and with the second operation they did the temporary ileostomy. She was in the hospital for a couple of weeks. Meanwhile, I was still working, taking time off work to help care for her, but had a family at home… I had two young kids and a husband. So it was, it was a lot.*

*(Participant 8)*

Similarly, another “sandwich” caregiver detailed the planning she undertook to look after her own family and the patient with cancer:

*Obviously, I had to juggle the children. That was the main concern. Two were in middle school and then two in elementary. So, it was a question of having to make sure that they were ready for school, and then someone picking them up at the end of the day…*

*(Participant 38)*

For some participants, the juggling act turned into a treadmill workout as they constantly organized and managed their responsibilities and made contingency arrangements, all the while considering the trade-offs entailed in attending to one set of responsibilities over another. For example, one caregiver (Participant 10) described working 8, 10, and 12-h days and then spending hours at the hospital before going home to look after her children’s meals. 

Many of the patient-participants acknowledged that their adult children had reorganized their employment and personal lives to care for them. A patient reflected on how her adult children provided generous care after her surgeries: “I would imagine they were trying to figure out how they could restructure their own family lives so they could be there when I need[ed] them, you know, take the time off [work] make sure I’ve got proper groceries” (Participant 7). Caregivers with ill spouses discovered that not only were they juggling employment and provision of care, but they were also managing all aspects of a household that had previously been shared.

Most caregivers commented on the challenges of constantly driving patients to appointments, surgeries, and other procedures. For caregivers in large urban centres, the driving task required scheduling time off work and devoting a large portion of the day to post-treatment care. Families outside urban centres faced the necessity of distance travel to receive treatment. A woman who cared for her husband (Participant 57) described a 4-h round trip for him to get chemotherapy. Caregivers who lived in rural locations and drove long distances to appointments, surgeries, and procedures often discovered there was no place for family to stay overnight, and therefore chose to drive back and forth instead. Participants in smaller cities could sometimes be assigned treatment in a specific location other than their home. A wife who drove a long distance to get her husband to treatments shared how driving in a large city was the most stressful task, second to worrying that her spouse’s cancer had metastasized (Participant 19). Travel was such an issue that some patients moved into the home of other family members temporarily while they underwent treatment, which could also put a strain on relationships. 

In their interview, one of the health professionals reflected on the challenge these caregivers faced in trying to find the optimal way to juggle these competing demands. “I feel like they’re working towards a finish line and then they’re not, and that’s disappointing” (Participant 68). Although the intensity of juggling employment and other responsibilities appeared to settle as patients completed treatment, due to recurrence, long-term effects of surgeries, and the emotional aftermath, the challenge seemed never completely finished for either caregiver or patient. 

### 3.2. Struggling with the Financial Impact

Throughout the interviews it was apparent that the caregivers often struggled with the financial impact of caring for a person with CRC. Financial reversals occasioned by CRC could last for years, and for some caregivers these constituted permanent changes, because treatment options and outcomes could extend over long periods of time. Because CRC meant that the ill family member could no longer work in many instances, couples and families previously accustomed to a dual income were forced to adjust to the constraints of one income. A wife, like other caregivers in the study, explained:

*You may have made really good smart moves financially, but all of that gets on shaky ground…it’s taking on all of the financial responsibility cause they’re no longer able to work. At that point he [husband] didn’t have a pension of any kind at all… Loss of income, huge additional expenses, all of that piece… So, there’s all of the financial responsibility.*

*(Participant 5)*

Even participants with more resources were aware of the financial impact and commented on how families with lower incomes would be adversely affected:

*We were financially stable enough that I’ve been able to do this [care for her mother] thus far. But I can imagine there are people that won’t be able to do this…. And, what does that look like trying to navigate?*

*(Participant 64)*

When the person who became ill was the major earner in the family, the caregivers not only struggled with the emotional and employment-related issues but also were faced with a more challenging financial reality. As a wife whose high-earning husband became ill said, “You really have to retool your life.” Compounding his reduced income was the fact that, in becoming her husband’s caregiver, she was forced to sacrifice her own financial and career advancements, and yet she and her husband also faced increased costs.

*You’re also living on one income. I have professional obligations. I mean, I can take time off, but there’s a lot going on… It just appalled me. I don’t, I don’t think people understand the financial pressure cause handing me a photocopied list of resources, almost all of which are fee for service. It’s frankly not helpful.*

*(Participant 5)*

The ongoing financial repercussions were especially daunting for the caregivers with children. A mother who lost her job while caregiving, and worried about her husband possibly dying, talked about the difficulties of parenting children and maintaining a household on her own:

*Now all of it’s on my plate. I’ve gone from being one of two people bringing in income to being the sole breadwinner. I’m also the person who takes care of the household chores. I’m the one who pays the bills. I’m talking care of the kids full-time… we couldn’t afford daycare anymore… I’ve had to take out loans and borrow money from family members… so—the financial situation and dealing with having small kids, that was an ongoing thing.*

*(Participant 59)*

#### 3.2.1. Responding to Financial Demands at Various Stages of Life

To cope financially, many participants commented on retirement as the preferred stage of life to deal with cancer. The participants commonly acknowledged that their life-stage (i.e., working-age versus retirement-age) was intimately related to the financial demands of caregiving. Like several participants, a woman whose husband was her caregiver during treatment talked about how she was fortunate he had retired:

*I think I was lucky that [husband] was retired. I don’t know what it would have been like if he did work because he was self-employed and he put in long hours and it was high, it was high stress… A lot of times he was wound right up when he came home from work. So, I wouldn’t have fared as well I can assure you, if he’d had to be working.*

*(Participant 26)*

Numerous participants framed retirement as enabling the caregiver to be constantly available to the patient with CRC. There were caregivers who remarked on how fortunate they were to have, for example, a disability plan or fewer financial concerns than other families. However, even for these participants, the uncertainty of CRC transformed the shape of their retirement, as described by a woman caring for her husband:

*Luckily, we were okay financially… he got disability and it was about the same, pretty close to the same of what he made, without income tax taken off. So, when I retired, of course my income went down too and he wasn’t supposed to have cancer. You know, like it was supposed to be normal, but he got the second bout of cancer after I retired.*

*(Participant 57)*

If retirement with generous pensions was the ideal stage of life to deal with CRC, the next best financial situation was having a caregiver with stable and flexible employment or nearing retirement with a kind employer. A woman in her thirties whose parents acted as caregivers commented on how cancer affected her father’s well-established employment; however, she qualified her situation as well-resourced, aware other families may have far different experiences:

*I know my dad certainly took way more time off, especially during periods where I was hospitalized, than he would normally take in a year. And then we also just did a lot of like financial, like, stretch and strain, so things were definitely more strained, but ultimately remained pretty stable. I think at the end of the day, I’m a very privileged person. I’m white. My dad is a professional, [he] has a very stable income.*

*(Participant 22)*

A husband who cared for his wife described how his employer made it possible for him to attend appointments, chemotherapy treatments, and be with her “every step of the way.” The participant was approaching retirement and indicated that if his boss had not approved alternate work assignments and arrangements, he would have retired to put his wife’s care first (Participant 31). 

In stark contrast, the caregivers who were younger, employed, or without savings or a financial safety net, described that few financial options or sources of assistance were available. One of the most poignant examples came from a young mother whose husband was receiving treatment, and who commented on her precarious finances in relation to her stage of life:

*It feels like when you’re older you’ve established, you’ve built a savings account and whatever, that’s one thing. But when you’re young and you still have little kids, and you’re still building your career, we didn’t have the safety net that somebody in their sixties would have.*

*(Participant 59)*

This participant further described worrying that she would be unable to pay their mortgage and feared losing their home, and yet she was unaware of anyone with whom she could discuss finances and of any sources of assistance.

*That was part of what really overwhelmed me okay, so my kids are dealing with their dad going through cancer and then, what’s going to happen? We’re going to get our house taken away. Well, that’s just an added stress. [It] would be really nice if there was somebody to at least go, hey, if you need help or have questions about financial planning, this is a great place to call.*

*(Participant 59)*

#### 3.2.2. Facing the Spectre of Lifelong Expenses

Following primary CRC treatment, families were often shocked to learn expenses continued to accrue. Even though many participants were fortunate to have good employer-provided health insurance plans, there were additional costs for equipment, diets, and medications that fell outside public and private plans. As one woman who cared for her husband explained:

*If you are of a low income, you possibly would get some things provided but to get the best things, it’s really expensive… he’ll put in an order, it’ll be $600. We’re fortunate that when he retired, we bought the best insurance…the means actually give you your life back.*

*(Participant 28)*

In instances where the patient required an ostomy, all the patient and caregiver participants commented on the high out-of-pocket expenses of supplies. Several also described the prohibitively high costs of nutritional supplements or foods that helped regulate ostomy output. Healthcare providers similarly described the significant cost of specialized ostomy supplies, especially if the patient had complications with their ostomy and/or hoped to return to work:

*When a person does get an ostomy, and they’re getting that first initial order of supplies, there can be some sticker shock when people see what that first bill may be. It can be anywhere from $150 to sometimes $500, depending on the type and the amount of ostomy products and supplies… And if a person is able to return to work shortly after having this type of surgery, there can be physical restrictions—especially for the first couple of months—that may inhibit someone who has a more physical type of job from being able to return to work.*

*(Participant 67)*

Other healthcare participants talked about incontinence and ileostomy supplies, and specific devices such as transanal irrigation systems, which are “astronomically expensive” and often not covered by the government-funded health care system or private insurance plans. Both the patients and the caregivers expressed frustration that they would be paying these medical expenses indefinitely. It was the same for medication costs. Health professionals often encountered families who said, “we cannot afford this and if I paid for this [medication], then I don’t have enough money for food” (Participant 76).

The caregivers and patients reported there were numerous forms required to access financial assistance and government health and social benefits beyond what was covered by public health care. Several caregivers commented that social workers had been extremely helpful for guiding them through documents for employer health insurance claims, tax breaks, and other government benefits. A social worker emphasized how a family’s resources always affect the quality of living and dying with cancer: “You’re not able to really focus on meaningful moments when you’re not sure how you’re going to be paying that month’s rent” (Participant 74).

## 4. Discussion

This study adds to the small but growing body of evidence describing cancer-related occupational and financial challenges facing caregivers, even in a universal government-funded health care system. Our findings point to temporary and long-term occupational and financial setbacks associated with CRC management and caregiving. Caring for a person with CRC involves managing occupational implications, including revamping employment arrangements, and juggling work, family, and household demands. Caregiver financial struggles include responding to financial demands at various stages of life and facing the spectre of lifelong expenses. 

Much of the research highlights reduced work or loss of employment as an indirect cost contributing to caregiver financial burden [18,35]. While our study was no exception, practical and emotional challenges among caregivers also arose from the complex and ongoing process of negotiating alternate work assignments and arrangements, taking paid sick or vacation days, or taking unpaid time off. The caregivers described feelings of loss and grief when they did not meet professional goals, they were no longer engaging in meaningful work, and their professional identity waned. Some described cancer caregiving as a turning point with long-term occupational, employment, and professional consequences, wherein caregivers had not been able to return to their rewarding careers. This aligns with previous research demonstrating that caring for a husband with an advanced illness presented challenges to a caregiver’s occupational life including diminished productivity, decreased quality of work, and missed opportunities for promotion [36]. The stresses of revamping employment arrangements, feelings of loss and grief, and inability to engage in meaningful employment perhaps contributes to emotional distress, caregiver burden, and poorer quality of life beyond these financial implications. These findings suggest that the impact of caregiving on occupational life can be significant and therefore provide potential targets to consider as CRO measures continue to be conceptualized and operationalized in the context of CRC. 

There were also numerous caregivers in our study who were not in a position to reduce or make the kinds of changes to their employment that would have enabled them to fulfill caregiver roles and responsibilities. There appeared to be a compounding effect over time such that the more responsibilities a caregiver juggled, the greater the pressure to make trade-offs among equally important responsibilities, and the higher the risk for emotional distress that might build and persist. Further, not being able to take time off work in fact generated additional costs at times, such as to pay for childcare. Our findings suggest that assessment of caregiver challenges ought to include questions about balancing occupation and employment along with other responsibilities and finance-related emotional distress and exhaustion throughout the cancer trajectory. These findings provide evidence to support the content validity of including occupational and financial items on CRO measures for cancer. However, efforts are needed to determine how to feasibly embed such CRO measures into care processes in a manner that enables healthcare providers to respond to identified caregiver needs and link caregivers to appropriate resources. 

In our research, it appeared that some caregivers of patients with CRC were perhaps at high risk of financial hardship and its associated distress and reduction of quality of life. Serious financial implications appeared to be greater in instances where the caregiver was younger, and faced also with juggling employment, looking after children, and assuming family financial responsibilities. This heightened risk has been suggested in prior studies where younger caregiver age, combined with greater patient symptom severity, was associated with higher financial burden [37,38,39]. In line with research on CRC survivors [40] and people with an ostomy [41], caregivers of a person with an ostomy in our research also described significant, often unexpected, and ongoing expenses associated with stoma management (e.g., ostomy supplies, nutritional supplements), placing them at higher risk of ongoing financial burden. Patients with metastatic or advanced cancer require resource-intensive therapy and care that contribute to financial hardship [35]. Further, individuals who are uninsured and low income, and from racialized or ethnic minority groups are more likely to have metastatic disease and to be more susceptible to financial burdens [35]. Our study included caregivers of patients with metastatic or advanced CRC, although we did not separate out their accounts during analysis given the smaller numbers in our sample. This therefore remains an area worthy of further study in the context of Canadian families with CRC [40].

The caregiver accounts in our study, including comments from those who were struggling financially and those who had access to adequate resources, clearly confirmed that there were financial challenges across contexts. This observation adds to the growing evidence of cancer-related financial hardship shouldered by cancer patients and their families in countries with government-funded or mixed government and privately funded health care [11,12,13,14,15]. Specific to CRC, in an Irish study, where there is a mixed public-private health care system, financial burden was ranked as highly as health burden among caregivers [28]. Indirect costs of caregiving appeared to drive the financial challenges in our study, pointing to the need for further investigation into the role of caregiver employment, including consideration of social support services and ways in which practice and policy interventions might temper caregiver burden. Notably, due to the nature of the sample we were able to recruit, our findings do not represent the more severe implications for those with serious employment or financial precarity. Research is urgently needed to understand the challenges, financial and other, of caregivers of people with cancer who experience serious socioeconomic disadvantage and structural vulnerability (e.g., poverty, unemployment, homelessness, racism, criminalization due to illicit substance use, or mental health stigma). In a 2020 Canadian study investigating family caregivers’ experiences caring for structurally vulnerable populations at end-of-life, caregivers themselves were found to be living within the constraints of structural vulnerability, with caregiving shaped by contexts of poverty and substance abuse, housing instability, and emotionally challenging relationships [42]. The authors of that study contend that the relatively minimal, one-size-fits-all caregiver supports that exist in Canada hold little relevance for vulnerable populations. Achieving health and health care equity in cancer care will require heightened consideration of the intersecting ways in which social and structural forces constrain caregiver agency and opportunities, and amplify vulnerabilities for poor health and social outcomes [43,44]. 

While several study participants commented on the important role healthcare providers played in facilitating efforts to obtain financial assistance, of concern were the reports of caregivers who felt utterly alone in navigating their financial reality and did not receive any guidance or resources from healthcare providers. Currently, there are no consistent approaches across Canadian cancer centres and elsewhere to determine financial challenges or related distress among patients and/or their family caregivers, including through the use of CRO measures [12]. Although some contend that discussions at the beginning of the cancer journey are most common [12], this timing would not take into account the financial picture that unfolds over time in the trajectory of CRC. Our research signals the importance of healthcare providers assessing not only patient but also caregiver financial challenges across the cancer trajectory. 

Routine assessments that include the use of CRO measures are perhaps one important strategy given evidence of the benefits of patient-reported outcome use in oncology. Evidence suggests that patent-reported outcomes influence patient-clinician communication by increasing symptom awareness, prompting discussions, streamlining consultations, and facilitating interprofessional communication [45]. Further, incorporating patient-reported outcomes into routine oncology care improves patient outcomes, quality of life, survival, and health system outcomes [46,47,48,49,50,51]. Though assessment alone will not ameliorate issues for caregivers, CRO implementation, including those that pertain to occupational and financial challenges, represents one aspect worthy of further investigation. Perhaps of even greater importance is referral to those who can offer relevant information, supports, and resources. However, information, supports, and resources specific to occupational, employment, and financial challenges are likely inadequate in many oncology contexts, signaling the need to develop accessible supports. Much work is needed to build the capacity of cancer organizations to make such CRO assessments and plans part of standard care, to enable healthcare providers to engage in discussion of issues related to finances, and to ensure robust supports and resources are available for patients and families. Clinically embedded research will be key to understanding how best to implement CRO in oncology care that link to necessary supports. 

### Limitations 

Strengths of this qualitative research included the large sample size and the diversity in participant experiences that enabled us to look for variation in our data as well as the inclusion of caregivers (spousal and non-spousal), patients, and healthcare providers. Unfortunately, we did not collect individual or household income data for caregiver or patient participants, making it difficult to characterize the sample across income quartiles. However, it was clear from participant accounts that individuals who were struggling financially as well as those with greater resources were represented in this study, and that they were all mindful of the financial implications that CRC occasioned. Limitations of this research also included our reliance on online recruitment methods that required participants to initiate contact with the study team. We intended to purposefully recruit caregiver and patient participants through oncology and community health care settings to obtain a sample of individuals who represented diversity in participant characteristics and experiences. However, the full diversity of sampling originally intended was not possible owing to the COVID-19-related restrictions and our reliance on online recruitment; therefore, the final sample was one of convenience. Our findings cannot represent the full diversity of caregiver and patient experiences, particularly those who experience greater socioeconomic disadvantage and structural vulnerability, who could not have been reached via online recruitment. Moreover, while we attempted to recruit male-identifying and gender-diverse caregivers, these individuals did not contact our research team as readily as female-identifying caregivers, and so a gender-based analysis of occupational- and financial-based sequelae was not conducted. Further research focusing on gender is warranted considering evidence that participation in the labour force is affected by caregiving to a greater extent among women than men [52]. Because interviews were conducted virtually, in the first 8 months of the COVID-19 pandemic, it is also possible that participant recollections were reflective of and shaped by pandemic-related public-health measures and stresses, particularly as they related to occupational and financial concerns. 

## 5. Conclusions

There have been calls for the routine collection of patient-reported finance-related outcome measures [53]. Given that caregivers of a patient with CRC face substantial occupational and financial challenges that last throughout the cancer trajectory, assessment of related CROs should also be considered. Further research is necessary to develop appropriate CROs to measure the multifaceted nature of the challenges CRC caregivers face and to determine how such assessments might best be integrated into oncology care delivery processes. Another important step will include initiatives that build capacity within cancer care organizations to embed occupational and financial assessments into care and to respond to caregiver needs via the offering of appropriate information, supports, and resources. 

## Figures and Tables

**Table 1:** Caregiver and Patient Participant Characteristics.

	Caregiver (*n* = 25)	Patient (*n* = 37)
Characteristic	Number	Number
Mean Age (years)	55	65
Gender		
Woman	22	16
Man	2	21
Non-Binary	1	0
Relationship to the patient (You are the patient’s…)		
Husband/Man Partner	1	
Wife/Woman Partner	15	
Non-binary Partner	1	
Daughter	6	
Son	1	
Friend (Woman)	1	
Relationship to Caregiver (You are your caregiver’s…)		
Husband/Man Partner		19
Wife/Woman Partner		8
Mother		1
Father		1
Sister		1
Daughter		1
Cousin (Man)		1
Friend (Woman)		2
Listed more than 1 caregiver role		3
Marital Status		
Married/Common-law/Living together	20	27
Divorced/Separated	1	4
Single	4	5
Widowed	0	1
Living Arrangement		
Living with the patient or their caregiver	16	29
Living alone	5	7
Other	4	1
Employment Status		
Full-time	8	6
Part-time	5	6
Not employed	11	23
Other	1	2
Cancer Stage of Patient		
1	3	2
2	1	10
3	6	16
4	8	4
Unknown	7	5
Patient Colostomy and/or Ileostomy		
Yes	11	18
No	14	19

## Data Availability

The data that support the findings of this study are not available on request from the corresponding author nor are they publicly available because they contain information that could compromise the privacy of research participants.

## Abbreviations

CRC: colorectal cancer; CRO: caregiver-reported outcome.
